# Novel Method for Determining Internal Combustion Engine Dysfunctions on Platform as a Service

**DOI:** 10.3390/s23010477

**Published:** 2023-01-02

**Authors:** Tomas Harach, Petr Simonik, Adela Vrtkova, Tomas Mrovec, Tomas Klein, Joy Jason Ligori, Martin Koreny

**Affiliations:** 1 Department of Electronics, Faculty of Electrical Engineering and Computer Science, VSB–Technical University of Ostrava, 17. listopadu 15/2172, 708 00 Ostrava-Poruba, Czech Republic; 2 Department of Applied Mathematics, Faculty of Electrical Engineering and Computer Science, VSB–Technical University of Ostrava, 17. listopadu 15/2172, 708 00 Ostrava-Poruba, Czech Republic; 3 Development Rear Lighting Electronics, HELLA AUTOTECHNIK NOVA, s.r.o., Druzstevni 338/16, 789 85 Mohelnice, Czech Republic

**Keywords:** cloud computing, exhaust emission testing and evaluation, new emission measurement methods, PaaS, quantile regression

## Abstract

This article deals with a unique, new powertrain diagnostics platform at the level of a large number of EU25 inspection stations. Implemented method uses emission measurement data and additional data from significant sample of vehicles. An original technique using machine learning that uses 9 static testing points (defined by constant engine load and constant engine speed), volume of engine combustion chamber, EURO emission standard category, engine condition state coefficient and actual mileage is applied. An example for dysfunction detection using exhaust emission analyses is described in detail. The test setup is also described, along with the procedure for data collection using a Mindsphere cloud data processing platform. Mindsphere is a core of the new Platform as a Service (Paas) for data processing from multiple testing facilities. An evaluation on a fleet level which used quantile regression method is implemented. In this phase of the research, real data was used, as well as data defined on the basis of knowledge of the manifestation of internal combustion engine defects. As a result of the application of the platform and the evaluation method, it is possible to classify combustion engine dysfunctions. These are defects that cannot be detected by self-diagnostic procedures for cars up to the EURO 6 level.

## 1. Introduction

Road traffic is considered one of the major sources of air pollution. It is a source of toxic compounds such as aromatic hydrocarbons, lead, CO, NO${}_{x}$, and particulate matter [1,2]. According to [3] passenger cars account for 44% of greenhouse gases produced by general road transport. Based on [4], road transport emits 42% of total NO${}_{x}$ emissions, 47% of CO emissions and 18% of PM emissions based on EU25 level. For the EU, manufacturers of Internal Combustion Engines (ICE) are legislatively forced to produce more efficient engines to reduce emissions. Observable trends include the deployment of direct fuel injection, overall combustion chamber volume reduction, start-stop systems, using turbochargers and many other components and new regulation and control proceses. Regulations and standards are transferred to the national levels of member states. The requirements to meet emission limits for newly manufactured cars are gradually increasing, see [5]. Emission limits are determined by Euro standards. At present, Euro 6 cars and lower emission standards prevail. These cars do not have to meet the strict requirements of EURO 7-e.g., Euro 7 rules ensure that vehicles are not tampered with and emissions can be controlled by the authorities in an easy way by using sensors inside the vehicle to measure emissions throughout the lifetime of a vehicle [5].

Currently, cars with a combustion engine defect are being operated and this allows relatively unlimited operation of the damaged car (e.g., sensors, intake leaks, engine leaks, injection system defects, SW modifications, etc.). The operator either does not know about the defect, or the performance or fuel consumption is not influenced to an extent that the engine will be repaired. Unfortunately, larger quantities of harmful emissions are produced in the mentioned case. Another problem is the expertise and limited skills of service technicians who can not properly analyse/repair serious engine management dysfunctions. Series-produced cars are measured as part of emissions measuring in Periodic Technical Inspections (PTIs) in static mode (steady load and engine speed). In these cases, the measured data is not continuously evaluated within the fleet. In addition, measurement under load in dynamic mode on a dynamometer using a dilution tunnel, is carried out as part of the development process for the purpose of reference and validation tests of combustion engines of cars (e.g., during the type-approval process). In the dynamic mode, cars are additionally tested in the mode of so-called “driving cycles”. The emissions tests mentioned are not initiated/motivated by the intention to measure a large number of cars and perform continuous complex analyzes using data from a large number of vehicles and the application of machine learning. The execution of the above-mentioned tests is not carried out in order to find defects based on a critical concentration of error manifestations from a large number of measured data.

The methodologies for vehicle emissions testing need to constantly reform and capture the real behavior of vehicles in real-world conditions. Emerging technologies can be used to enhance them. As the use of cloud processing platforms increases in various industrial areas, potential uses could be found in the automotive emission testing sector.

The objective of this research is to have the ability to determine whether the vehicle is being operated with an internal combustion engine fault, based on a combination of gathered information. In particular using the results of an original emission tests on a significant sample of vehicles and regardless of whether there is a fault stored in the memory of the electronic control unit. To achieve this, the data regarding an emission production (NO${}_{x}$, HC, CO, CO${}_{2}$, O${}_{2}$, smoke absorption value, lambda factor), vehicle mileage, engine volume, Euro class, Engine Condition State (ECS) are monitored. Our evaluation platform uses combined data from a significant sample of technical inspection stations (on a European level) and a common evaluation cloud with the application of machine learning techniques. The stations have at their disposal common diagnostic and measurement tools, determined by legislation in individual EU member states.

In the beginning of the article the proposed emission test is discussed thoroughly along with the proposed test setup. A static testing method using a chassis dynamometer and an emission analyzer was designed. This static method is based on a procedure of testing the engine at certain engine speed and engine load, until the value of produced emission compounds stagnates. According to [6] instantaneous emission measurements on chassis dynamometers are becoming increasingly common for car-makers and for environmental emission factor measurement and calculation. The dynamics of gas transport in both the exhaust system of the car and the measurement system last significantly longer than 1 s and can introduce considerable inaccuracy into the continuous measurement. Above described method can compensate for this phenomenon.

This section is followed by the experimental part, in which the relevance of the proposed test is analyzed. The emission measurement investigates the relationship between the power produced by an internal combustion engine and the various emission compounds produced in exhaust gases. The next section deals with the proposition of the use of cloud processing platform. The developed topology of the evaluation technique is focused on gathering emission data from many testing facilities. The data processing is presented in the next segment. The solution uses quantile regression models to evaluate a simulated data set and ultimately provides information to distinguish vehicles, with any variations related to emissions production. The outcome of the article is a finalized proof of concept.

The whole methodology can be tested using real vehicle data. The aim of future work is to establish experimental testbeds for new measurement procedure. The data collection and evaluation method can be deployed using the concepts described in this article. Testbeds will implement this new method for emission evaluation with the idea of opening a discussion about the application at the EU25 level.

## 2. Background

The negative effect of road traffic-generated, tailpipe emissions on people’s health is, in general, indisputable. As an example, several epidemiological studies have found a definite association between airborne Particulate Matter (PM) and adverse health effects. Particulate matter is associated with pulmonary and cardiovascular diseases, while less clear traces lead to cancer and psychological problems. One epidemiological study even found that exposure to traffic-related air pollution during pregnancy and during the first year of life was associated with increased autism in children [7,8,9]. The direct emissions of motor vehicles also influence the global climate change phenomenon [10]. The primary emissions formed by road vehicles contain gaseous substances that form a secondary layer of aerosol, which is prone to oxidation [11]. Oxidized substances can change their chemical composition and become either more or less toxic [12].

As this article deals with a unique, new platform and an original emission evaluation technique for light-duty vehicles, only applicable emission evaluation procedures will be discussed. The direct emissions of road vehicles are measured with the help of standardized driving cycles that help to establish whether the vehicle is compliant with the most current emission test standards. New European Driving Cycle (NEDC) has been critiqued in the past for not adequately covering the entire operating range of a combustion engine [13,14]. This driving cycle, designed in the 1980s, is now considered outdated and not relevant because it is too uniform.

The worldwide harmonized light vehicles test cycles are part of the Worldwide Harmonized Light Vehicles Test Procedures (WLTP). The procedure of emission testing with WLTP is considered more relevant for simulating a real-world emission profile of a vehicle in laboratory conditions. A transition from NEDC to WLTP was made from 2017–2019. Along with WLTP, a Real Driving Emissions (RDE) test is required for road vehicles which fulfill EURO 6d-TEMP emission standards. This test is conducted with the help of a Portable Emissions Measurement System (PEMS), which provide very accurate real-time monitoring of the pollutants emitted by internal combustion engines (HC, CO, CO${}_{2}$, NO${}_{x}$ and PM). This methodology was developed because there were inconsistencies found between NO${}_{x}$ produced during vehicle operation on the road and during laboratory tests (Dieselgate 2015) [15]. The Volkswagen emissions scandal, also known as Dieselgate, erupted in September 2015 after the U.S. Environmental Protection Agency (US EPA) announced that German carmaker Volkswagen had equipped its diesel cars with software that detected that the engine was operating in exhaust test mode, and changed engine settings to temporarily reduce the amount of Nitrogen Oxides (NO${}_{x}$) produced, so that its vehicles met the legal limit.

### Literature Review

Emission measurement techniques and the subsequent evaluation of their values are the subject of constant research. The purpose of this publication is to point out the methodology of data evaluation, in order to reveal the dysfunction of the combustion engine. In already published literature, it is possible to find approaches for measuring and evaluating emission data from vehicle, which point to some useful data. In [16] a method was developed for the identification of vehicles with impaired functional performance of the particulate filter system. By using the developed method diesel vehicles with properly working after-treatment systems can be distinguished from vehicles with removed or malfunctioned particle filters. In [17] a new approach for static NOx measurement in PTI was developed, that can be used for detecting high emitting vehicles. The cause of high NOx production may be a malfunction of one of the power management components of the combustion engine.

It is possible to use vehicle emission and operational data for the purposes of emission production modeling. In [18] an approach was developed, where neural networks and regression analysis is used to predict fuel consumption and emission levels at different vehicle conditions. In [19] models are used to predict values of various emission compounds including nitrogen oxidex, hydrocarbons and fuel consumption based on instantaneous speed and acceleration levels of the vehicle.

Combined with the possiblities of remote cloud processing, vehicle emissions testing can be enhanced and emerging technologies can be used to improve them. Some researchers, such as mentioned in [20], propose using IoT or GSM-based, emission monitoring systems, where they combine multiple gas sensors with IoT solutions but do not propose further use or method for processing the data. A Networked Remote Sensing System (NRSS) described in [21], is built on the concept of exhaust gas remote sensing systems mounted on roadways to measure all passing vehicles. The concentrations of CO${}_{2}$, CO, HC, NO${}_{x}$ and particulate matter can be measured by RSS very quickly and, by utilizing cameras, high emitting vehicles can be identified. The NRSS proposes to interconnect measurement devices and provide a complex, real-time monitoring system to record emissions of many vehicles simultaneously.

As mentioned in [22] IoT is used to bring all emission data under one system to preform data analyses in the cloud. Their team also performed critical cloud processing tasks to predict future events of emissions. In [23] the authors propose building a new cloud framework for transportation systems for monitoring various aspects affecting the vehicle, including emission data. The application will be created for a project based on criteria stated in [24], which will help to find the right cloud system and data processing technique. The cloud system must run our own application on top of its platform, efficiently and supportive of our proposed third- party system, without compromising security. Article [25] describes how the vehicle to cloud service must be oriented to Software as a Service (SaaS) or Platform as a Service (PaaS) to handle big data. The big data mentioned in that article includes greenhouse gas emission data and service statistics; the two key components implemented in our application.

The research gap addressed by the described scientific activities is the absence of evaluation of vehicles with engine dysfunction based on accumulated information from a large number of cars. The solution provides a new concept of a test platform (PaaS) for the level of a large fleet and model utilization based on the principle of machine learning. Thanks to the tools of this methodology, it is possible to identify vehicles with increased production of one or more components of exhaust emissions.

## 3. Research Methodology

One of the goals of the new platform and the investigated emission test methods is to evaluate the relationship between the power produced by an internal combustion engine and the various emission compounds produced in exhaust gases. Therefore, a specific test procedure is proposed and described in more detail in the following section.

To achieve the possibility for evaluation of impact on a regional or national level, the testing and evaluation must be separated into multiple phases as follows:

1. test setup definition,
2. vehicle database establishment with respect to selected categories—vehicle parameters (vehicle type, volume of combustion chambers, type of fuel used, date of manufacture, types of exhaust aftertreatment systems installed and the engine systems condition),
3. fleet level evaluation.

### 3.1. The Emission Test Concept

The most important criteria in designing the emission test for evaluation of different engine emission emitting profiles are: (i) test repeatability and (ii) ability to compare different engine units. Because of the inability to determine the volume of emitted compounds during dynamic tests in the proposed type of vehicle testing facility, a static testing method had to be designed. By determining the usable power of the engine unit, a variety of static conditions can by simulated as a substitute for dynamic testing. The static emission method is conducted as follows:

1. Measurement of maximum torque (Nm), power (kW) of the engine unit, and establishing the engine speed (RPM) at which the 100% of maximum engine power occurs (testing point 3).
2. Calculation of 75% of maximum power at wheels (kW) and establishing the engine speed (RPM) at which 75% of maximum engine power occurs (testing point 2).
3. Calculation of 50% of maximum power at wheels (kW) and establishing the engine speed (RPM) at which the 50% of maximum engine power occurs (testing point 1).
4. Execution of emission test at specified engine speeds (RPM) and loads (%).

**Figure 1:**
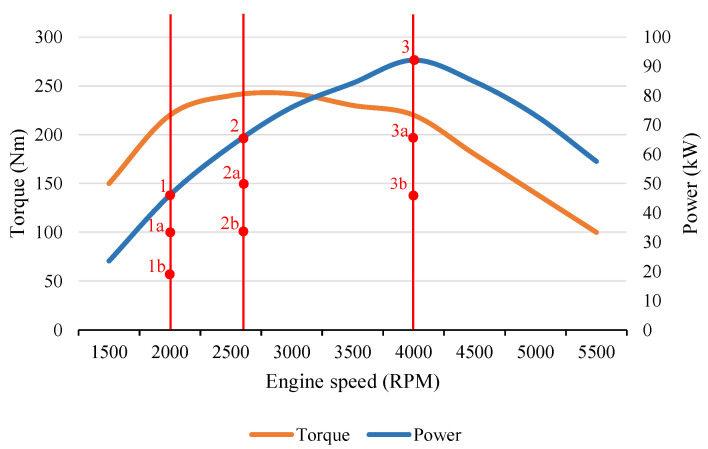
Testing points for the proposed emission testing cycle.

Various engine loads (50%, 75% and 100%) are tested at the calculated engine speeds to analyze behavior of the unit in different conditions for the purpose of determine emission compound formation. Testing points 3a, 2a and 1a capture measurements at 75% engine load. Testing points 3b, 2b and 1b then capture 50% engine load. Along with other data, these values of the measured emission components will later be used as input variables for the new evaluation method. Additional measuring conditions can be added in the order of pursuance to a certain emission component. Figure 1 captures the specific measured range of the proposed emission testing procedure. Every harmful emission compound has its own specific conditions for formation, so each measurable emission compound must be monitored separately. The accuracy class of the emission tester must be considered, and the measuring mistake must be calculated in the results. For the test results to be valid for each vehicle, the vehicle systems status and testing conditions must follow specific propositions.

### 3.2. Testing Procedure and Facility Concept

At a regional level, defining a testing procedure for a single vehicle is crucial for a solid foundation and reliable results to gauge EU fleet level impact on various emission compound formations. As was mentioned, the objective of this research is to have the ability to determine whether the vehicle is being operated with an internal combustion engine fault, based on a combination of gathered information. The idea is that the evaluation platform uses combined data from a significant sample of technical inspection stations (on a European level), using a common evaluation cloud with the application of machine learning techniques. By inspection technician are monitored and stored: vehicle mileage, engine volume, Euro class, ECS determined by the service technician (uses common service diagnostic devices and quick visual inspection, see Table 1). The stations have at their disposal serial diagnostic tool (OBD-II reader) and other measurement tools determined by legislation in individual EU member states.

All the systems of ICE that have the potential to have an impact on excessive emission compound formation must be tested. These are monitored by the “On-Board Diagnostic” system; however, an additional systems check may be required. This prefatory control must be conducted by authorized and trained personnel to maximize the relevance of the results. As the result of the prefatory control is one of the variables for the statistical evaluation, a sophisticated approach should be considered when evaluating vehicles for their system´s functional status. However, this is not within the scope of this article, so the assessment is simplified into ECS. It is graded 1 to 5, with 1 being the best possible grade for the system´s operational status. This grading system separates the emission measurement data that is used for establishing the emission production profile for vehicles with similar parameters (with grades 1 and 2). Vehicles with grades 3 and worse are the ones that the evaluation method is designed to search for in its application. Hereafter, this variable will be referred to as the ECS. For petrol ICEs, the functionality of the oxidation catalyst must be evaluated, the fuel metering system and the λ control system and others depending on the engine type. For diesel ICEs, the correct operation of the fuel metering, the charge air control, the correct function of the SCR catalyst and relevant processes and other installed exhaust after-treatment systems, must also be evaluated depending on the engine type.

The equipment of the systems for the suppression of selected emission components is determined by the level of compliance with EURO standards. The EURO 7 standard is currently being discussed.

The developed topology of the emission testing facility is focused on gathering emission data from many testing facilities and numerous vehicles using just a roller chassis dynamometer and basic emission analyzer, without the need for a dilution tunnel (device for creating a measurable emission flow sample in case of performing advanced emission analysis).

Steps of the testing procedure:

1. Standard service procedures are carried out for establishing ECS (Visual inspection, connection of OBD-II diagnostic interface: vehicle ID, reading of pending trouble codes, permanent trouble codes, readiness code and uneven engine run check).
2. The vehicle is tested on the chassis dynamometer. The dynamometer creates a negative traction force for tested vehicle (the measuring points are described in the Section 3.1). Emission compounds (NO${}_{x}$, HC, CO, CO${}_{2}$, O${}_{2}$, smoke absorption value and lambda factor) are measured with an emission analyzer.
3. Measured emission compounds for individual load points, vehicle information (make, model, VIN, current mileage) are sent to the MindSphere application for processing.
4. The application, using the quantile regression model, determines the expected range of emission components and evaluates whether the emission systems of the tested powermanagement is fully functional or whether there is a defect within this system.
5. The test device receives from the cloud application the required dispersion of emission components for individual load points, on the basis of which the test technician makes a decision about the state of the systems that have an influence on the emission compounds formation.

**Figure 2:**
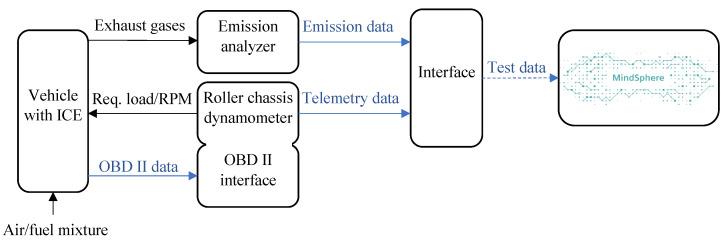
Concept for testing facility connection to MindSphere.

Figure 2 displays the concept fot the testing facility and the equipment needed for the proposed test. It also shows the data that need to be transferred to the cloud application for evaluation.

### 3.3. Testing Procedure and Facility Concept

The measurements in this article are submitted on a MAHA LP3000 roller chassis dynamometer, which allows a variety of engine parameter measurement approaches. The emission analyzer used for this emission test was the Bosch BEA050. The test vehicle was an Audi A3 with specifications shown in Table 2.

An engine ECU software modification was executed to capture the simulated behavior of faulty turbocharger regulations. A turbocharger faulty regulation can be caused by several factors. One very common factor is an excessive amount of soot contamination on the variable-geometry turbocharger elements and their surrounding mechanisms. This forms an obstruction which limits the actuation response of the variable geometry (the boost pressure is under-regulated or over-regulated). In the event of this fault, the engine management is in a limp mode, but many users accept limited performance and do not take steps to repair the fault right away.

**Figure 3:**
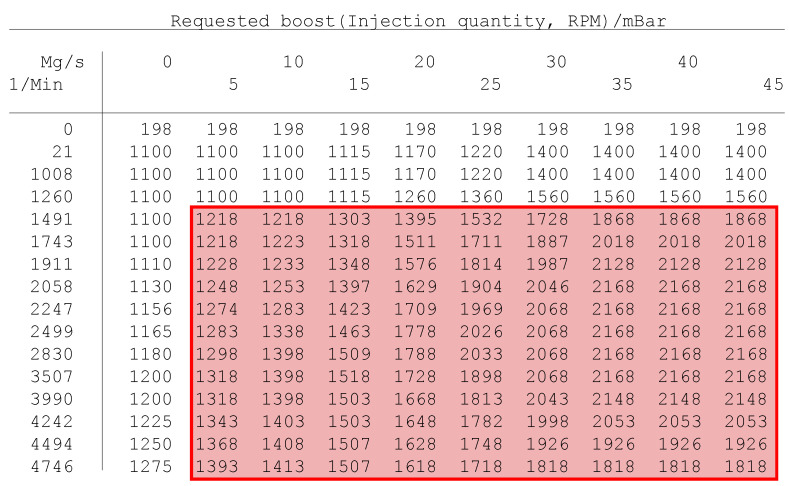
Value of requested absolute intake pressure (mBar).

This simulation’s purpose is to simulate the mechanical pollution of the turbocharger due to poor mixture formation and excessive soot production. The state of incorrect mixture creation (where an excessive amount of air is present) is simulated by changing the values stored in the FLASH memory of the ECU. The values of requested boost and all boost limiters were raised by 118 mBar. Example of the changed area of the memory is highlighted in Figure 3.

**Figure 4:**
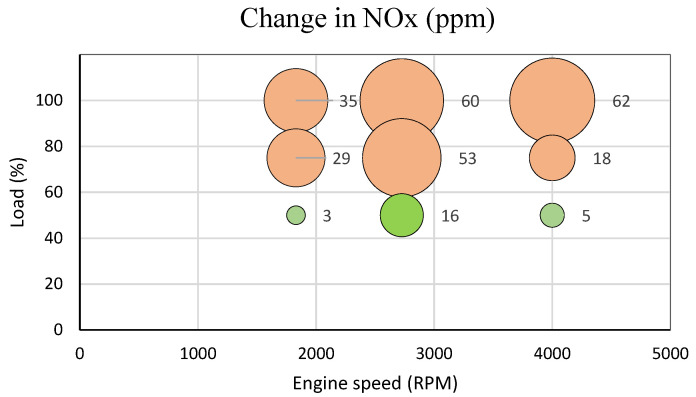
Change in NO${}_{x}$ values before and after simulated dysfunction.

As a result of the mentioned software modification, the state of influence of an excessive amount of air sucked in by the engine and entering the combustion process was simulated. By forcing more air into the engine, the ECU measures this condition through the MAF sensor and responds accordingly. The ECU then changes the setup of the EGR valve to introduce more exhaust gases to the combustion process. Overall, it can be claimed that this dysfunction has some impact on the emissions compounds formation (as seen in Table 3 and Figure 4), which shows the impact on NO${}_{x}$ emission values.

The experimental measurement indicates the possibility of detecting a certain engine management dysfunction via the acquired measurement data from the emissions test. Similarly, software modification can be used to simulate a number of ICE malfunctions. This will be the subject of follow-up research on ICE fault simulations.

### 3.4. Vehicle Emissions Profile Evaluation

This section describes the possibility for remote data collection to the IoT operation system on a cloud and unique measuring application. In a real-life application of the proposed method the data can be gathered during analysis in test facilities, as mentioned in Section 3.2. Each IoT platform offers various services. In the following paragraph, the initial evaluation of the IBM Watson and the Siemens MindSphere is described in the context of finding the right platform for this application.

IBM Watson cloud and Siemens MindSphere are standard IoT cloud platforms used in industrial applications. It is possible to develop original applications using their software tools (e.g., programmed in Python). IBM offers an IoT platform, node-red and cloud-object storage database. MindSphere offers a MindConnect IoT platform along with Mendix development application.

The emission processing application requirement is to be built specifically for its dedication along with a specific dashboard/user interface.

Using Mendix, it is possible to create a new application faster, along with the option of using MindSphere’s powerful connectivity. Thus, MindSphere has become our platform of choice. MindSphere is the PaaS application. It uses cloud services like AWS, which is very reliable, secure and always accessible with very minimal downtime, even though the data is collected at fleet level.

**Figure 5:**
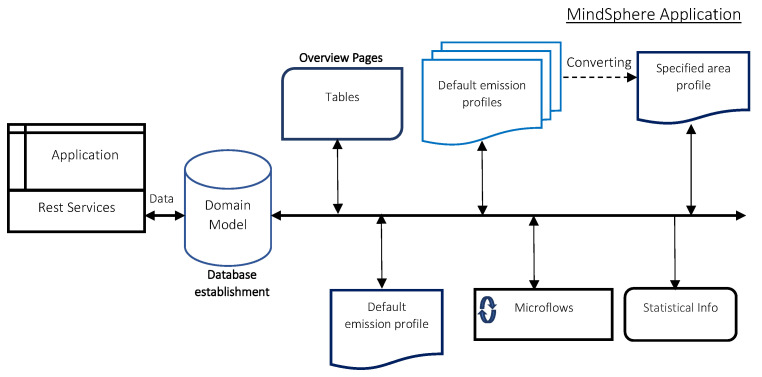
Concept for data evaluation.

**Figure 6:**
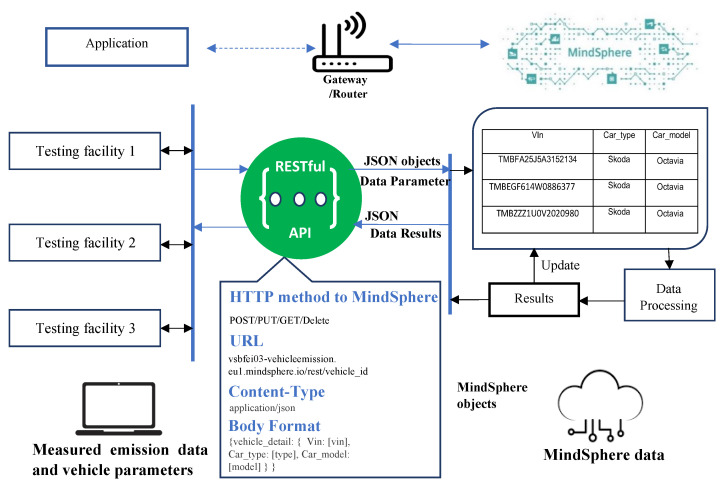
New platform for data collection.

The emission data processing, cloud application can be created on top of a framework native to Mendix IDE and MindSphere. The cloud application requirements include database creation, user interfaces and import/export of the measured data. The fleet level data collected in testing facilities can be sent through the REST API endpoint to the cloud application (as seen in Figure 5). Optional direct upload via spreadsheet is possible. Either way, in both cases, the data is imported/exported as a JSON variable. Then, the emission application binds JSON objects to Mendix objects (or vice versa) to do data processing and send results, respectively. The input data from a single vehicle can then be compared with data from the established emission profile database, as described in Section 4. Figure 6 represents the process of data collection and transfer, back and forth to the MindSphere and testing facilities.

## 4. Fleet Level Evaluation

For each testing point, three linear quantile regression models were created to model the 50% (median), 75% (Q75) and 90% (Q90) quantiles of the natural logarithm of emission compounds (NO${}_{x}$ measurement values were used throughout the paper as an example). The logarithmic transformation of the dependent variable was applied due to the high skewness of the emission compounds in all testing points. The independent variables were identical for all models, i.e., volume of engine combustion chamber (in thousands cm${}^{3}$), EURO emission standard category and mileage overall system condition (in tens of thousands km). All independent variables were considered as numerical with no transformation or standardization, except for the vehicle EURO emission class, which was considered as categorical. The variable (describing types of exhaust gas aftertreatment systems installed) was excluded from a set of independent variables due to its significant association with the EURO emission standard category. Inclusion of both variables would cause instability of regression coefficients. The set of independent variables could be modified once applied in real-world circumstances. The models were fitted using R software (version 4.1.1, rq function, quantreg package) and the significance level was set to 0.05 [26].

The advantage of using quantile regression is its robustness and resistance to outliers [27,28]. Furthermore, it does not make any strong assumptions about the distribution of independent or dependent variables or residuals. Obtained regression coefficients have clear interpretations and can provide valuable information about the importance of independent variables. Moreover, quantile regression allows us to predict the emission compounds (with the model for the median) and approximately determine acceptable upper bounds of emissions (with models for Q75 and Q90), based on specified vehicle parameters.

The dataset contains information about 9986 vehicles. Only those vehicles with lower ECS (that is, values 1 or 2) were used to build the models. Out of all such vehicles (4595 vehicles), 50% (2297 vehicles) were dedicated as the training set and used to build the models, 25% (1149 vehicles) were used as the first test set to assess the fit level of the quantile regression model for the median of the natural logarithm of the emission compounds (as an expected prediction model for the emission compounds), i.e., to display correlograms and calculate the adjusted coefficients of determination (R2adj) for each testing point. The rest (25%, 1149 vehicles) and vehicles with higher ECS (that is, values 3, 4 or 5, 5391 vehicles) were used as the second test set to evaluate the accuracy of the models with respect to our original goal; to use these models to detect vehicles with any of the engine subsystem malfunctions that have an impact on the monitored, produced exhaust emission compounds.

We expect a high proportion of the vehicles with 3–5 ECS to show real emission compounds of above Q75 and Q90. Such results indicate that it would be possible to use the models for preliminary detection of the vehicles, with possible engine subsystem malfunctions based on unexpectedly high emission compounds. Finally, a cut-off value was found for the minimal number of testing points, in which a vehicle must exceed Q75 and Q90 to be suspected of possible engine subsystem malfunctions with acceptable accuracy. For that purpose, the ROC curve was used. Overall accuracy, sensitivity and specificity were evaluated.

The description of the linear quantile regression models for the median of the natural logarithm of the emission compounds for each testing point is available in Table 4. Results show substantial stability of regression coefficients (except for the intercept), which suggests stable importance of the independent variables in modelling the emission compounds in all testing points. With a 1000 cm${}^{3}$ increase of the combustion chamber volume, we expect approximately a 2–4% increase in emission compounds in all testing points. Accordingly, with a 100,000 km increase in mileage, we expect approximately a 3–7% increase in emission compounds in all testing points. As expected, higher Euro Emission Standard category led to a decrease in emission compounds of approximately 50% for Euro 3, 75% for Euro 4, 82% for Euro 5 and 92% for Euro 6, in comparison with the baseline category of Euro 2.

**Figure 7:**
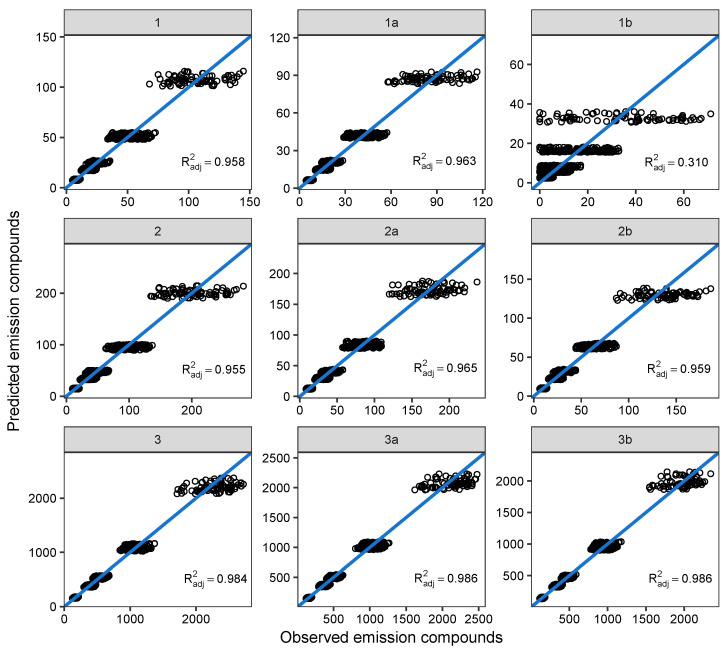
Correlograms present the goodness of fit of the quantile regression model for the median of the natural logarithm of the emission compounds as the potential prediction model for the values for each testing point. The adjusted coefficients of determination ($R_{\mathrm{adj}}^{2}$) are also displayed.

By applying exponential function to the predicted values for the first test set, we obtain predicted NOx values in original units. The goodness of fit is presented with the correlograms with adjusted coefficients of the determination displayed, see Figure 7. All testing points, except for the testing point 1b, show very good performance of the quantile regression models with adjusted coefficients of determination above 0.950. Further, the quantile regression model for the median is not used because we do not aim to build a prediction model for the emission compounds. However, by assessing its goodness of fit, we aim to verify the application of the quantile regression itself for estimation of the acceptable upper bounds of emission compounds.

Whether or not testing point 1b would have any significance (in the context of potential engine management dysfunction detection via NO${}_{x}$ measuring) is a point of further discussion, being that production is at its lowest across the entire testing range. If this were not the case for real world data measurements, additional independent variables would need to be added to the data.

All regression coefficients (except for the intercept) are very similar to the coefficients presented in Table 5, suggesting that the effect of each independent variable is relatively constant over the entire distribution of emission compounds in all testing points. Table 6 and Table 7 present the quantile regression models for the 75% quantile and the 90% quantile of the natural logarithm of the emission compounds.

The second test set is passed to the models and the proportions of vehicles (with a real value of emission compounds above Q75 and Q90) are determined for each testing point (see Table 8). As expected, the proportions corresponding to vehicles with ECS values of 1 or 2 reflect the definition of Q75 and Q90 (i.e., above these estimates, approximately 25% and 10% of the vehicles are in each testing point). The results show that the vehicles with ECS values of 2 contributed to this phenomenon, even though in the training set the ratio of vehicles with ECS values of 1 and 2 was balanced. Nevertheless, these results can also be perceived as another confirmation that the built models for Q75 and Q90 work as anticipated. The proportions corresponding to the vehicles with ECS values of 3, 4 or 5 increase with a higher coefficient (i.e., almost 100% of vehicles with ECS values of 5 were above Q75 in each testing point (except for in testing point 1b).

Table 9 presents the vehicle distribution to testing points ratio for which vehicles from the second test set had emission values higher than Q75 and Q90 (with respect to their ECS).

**Figure 8:**
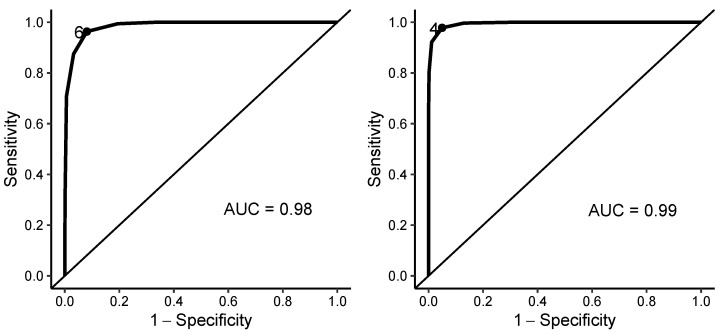
Analysis of the prediction ability of the number of testing points in which the real value of emission compounds exceeded Q75 (**left**) or Q90 (**right**) to be suspected of possible engine subsystem malfunctions with ROC curves (AUC = area under the ROC curve).

Finally, the optimal cut-off value is found for the minimal number of testing points in which a vehicle must exceed Q75 or Q90 to be suspected of possible engine subsystem malfunctions (see Figure 8). The optimal cut-off value for the classification based on the model for Q75 is 6 (i.e., if the real value of emission compounds exceeds the estimate of Q75 in at least 6 testing points, the vehicle should be seriously suspected of possible engine subsystem malfunctions). The overall classification accuracy for the cut-off value 6 is 95.6%, the sensitivity is 96.3% and the specificity is 91.9%. The optimal cut-off value for the classification based on the model for Q90 is 4 (i.e., if the real value of emission compounds exceeds the estimate of Q90 in at least 4 testing points, the vehicle should be seriously suspected of possible engine subsystem malfunctions). The overall classification accuracy for the cut-off value 4 is 97.3%, the sensitivity is 97.8% and the specificity is 95.0%.

The results suggest that the prediction ability of the number of testing points in which the real value of emission compounds exceeded either Q75, or Q90, is comparable. The overall accuracy, sensitivity, and specificity were slightly higher for the classification based on the model for Q90. Even though the performance of the quantile regression models for testing point 1b was clearly unsatisfactory, the presented approach (based on the combination of the information from all testing points), is not sensitive to the effects of possible inaccuracies caused by one testing point with low significance for overall results.

## 5. Discussion

In Europe, there are a large number of vehicles that are operated with a combustion engine dysfunctuon that affects the production of emission compounds. Some defects in emission systems cannot be detected by the OBD-II on-board diagnostic system or by means of a mandatory periodic technical inspection. Emission testing during PTI is different from homologation emission tests and only a part of the emission components are measured (e.g. only smoke absorption coefficient is often measured in diesel engines; internal car emission systems for NO${}_{x}$ reduction not need to be tested). The measurement is aplied by unloaded engine (at idle speed, increased idle speed, overrun speed). The proposed method is based on the measurement of emission compounds, much the same as in homologation tests. The chassis dynamometer enables to generate negative tractive force for tested vehicle and measure the vehicle under higher loads that correspond more to normal use. Due to the possibility of stabilization of values of emission compound under static load, continuous measurement with a regular emission analyzer is more accurate and reliable than during short dynamic tests of an unloaded internal combustion engine. The uniqueness lies in the comprehensive service of the platform, which evaluates the measured emission components including vehicle information in a cloud application. Statistical operations are carried out within the framework of comparison with historical data of the same or very similar vehicles. The test equipment obtains the required dispersion of emission components for individual load points, and the technician it can be clearly evaluated whether the vehicle is being operated with a defect that affects emissions.

The emission cloud application is created on PaaS and has various advantages for further expansion of the data processing application. Some of the benefits are simple sharing of emission data among different applications created within PaaS and remote access to the data and high computing power to run the model irrespective of current system condition. This prototype application is part of a new approach to a methodology of emission data measurement and processing.

The evaluation concept follows an analysis of a single vehicle in comparison with the fleet level dataset. The measurement could be then displayed to the testing facility technician to indicate whether the vehicle fulfills the expected produced values of exhaust emission compounds with respect to a vehicle’s features.

Statistical modelling of exhaust emission compounds was based on quantile regression, which used predefined vehicle parameters for estimating the median (the 75% quantile and 90% quantile of emission compounds), to predict the emission profile and determine approximate, acceptable upper bounds of emission compounds. The optimal cut-off value was found for the minimal number of testing points in which a vehicle must exceed the estimates of the 75% quantile or the 90% quantile of the emission compounds to be suspected of possible engine subsystem malfunctions. The results suggested that if a vehicle exceeds the estimated 90% quantile in at least 4 testing points, it should be seriously suspected of engine subsystem malfunctions. The overall classification accuracy based on this approach reached 97.3%.

As previously mentioned, the data for evaluation is generated. The data generation imitates, to some extent, the general dependencies of the measured emission compounds with the vehicle´s EURO emission class, mileage, installed emission aftertreatment systems and others. The set of these independent variables could be modified once applied in real-world circumstances. The aim of future work is to establish experimental testbeds for measurement and data collection. These will implement this new method for emission measurements. The gathered data will create a database of valid samples from real-world, vehicle emissions.

In this phase of the research, real data was used, as well as data defined on the basis of knowledge of the manifestation of internal combustion engine defects. The dataset created in this way is used for the first phase of research and verification of the proposed method and developed algorithms. The subsequent phase will exclusively use real measured data.

It is also possible to use the methodology described in the article in an extended version, in which a specific detected defect would be assigned to the detected excessive emission production (after the inspection of the vehicle by a diagnostic technician). With this step, it would be possible to introduce additional parameters about the condition of the vehicle into the database of measured vehicles, which would help to determine not only that the vehicle is being operated with an engine fault, but also what specific fault it is.

## 6. Conclusions

A real-world vehicle fleet is comprised of vehicles with high variance of maintenance schedules and previous operational conditions, these can highly influence the production of various exhaust emission compounds. The proposed method for emission testing and data processing provides a framework for emission compound prediction based on the parameters of an individual vehicle. Although the proposed measurement technique is laboratory-based, it captures the behavior of an engine unit at a variety of engine speeds and loads. The proposed methodology has the goal of detecting the vehicles with any of the engine subsystem malfunctions that impact the level of monitored emission compounds produced.

Currently, new concepts of E/E architecture of cars are being developed, enabling the implementation of sophisticated telemetry operations, including the provision of information to the cloud. These are 2030+ architectures (they use a service oriented gateway or a high performance computer). Therefore, new power management services for the internal combustion engine can be assumed. Provided that emission diagnostic services/algorithms are implemented, vehicles can provide information about the current status of emission systems directly to the cloud. That is, without the need to use emission testing within PTI. Therefore, the research described in this article is a preliminary step for applications of future cloud services focused on the online evaluation of combustion engine dysfunctions. Our cloud application and researched algorithms can be implemented within new architectures using PaaS.

## Figures and Tables

**Table 1:** ECS determination principle.

Type of Defect	Value	ECS
OBD-II-Readiness code	1/0	All 0 → 1 1×1 → 2 2×1 → 3 3×1 → 4 4 or 5×1 → 5
Uneven engine run	1/0	
OBD-II-Active pending DTC	1/0	
OBD-II-Active permanent DTC	1/0	
Visual engine state	1/0	

**Table 2:** The testing vehicle parameter list.

Engine BXE, 1.9 TDI, Audi A3, Hatchback, Manual			
Emission standard	EURO 4	EGR	YES
Smoke absorption value	1.5 (m${}^{-1}$)	Catalytic converter	YES
CO${}_{2}$ Average	135–138 (g/km)	Turbocharged	YES
CO	0.131 (g/km)	Intercooler	YES
HC	x	Lambda regulation	NO
NO${}_{x}$	0.224 (g/km)	DPF/FAP	NO
HC NO${}_{x}$	0.248 (g/km)	Engine displacement (cm${}^{3}$)	1896
Particles	0.02 (g/km)	Power output (kW/RPM)	77/4000
Injection system	Unit injector	Torque (Nm/RPM)	250/1900

**Table 3:** Emission measurement results.

NO${}_{x}$ Emissions (ppm)	Before Dysfunction			After Dysfunction		
Engine Speed (RPM)	50 % Load	75% Load	100% Load	50 % Load	75% Load	100% Load
1830	179	375	1895	176	404	1930
2726	169	369	1662	153	422	1722
4000	166	315	1688	161	333	1750

**Table 4:** Sample NO${}_{x}$ data for Testing Points (TP).

Eng. Disp. (cm${}^{3}$)	Veh. EURO Class	ECS	TP 1 NO${}_{x}$ (ppm)	TP 2 NO${}_{x}$ (ppm)	TP 3 NO${}_{x}$ (ppm)	TP 1a NO${}_{x}$ (ppm)	TP 2a NO${}_{x}$ (ppm)	TP 3a NO${}_{x}$ (ppm)	TP 1b NO${}_{x}$ (ppm)	TP 2b NO${}_{x}$ (ppm)	TP 3b NO${}_{x}$ (ppm)
1598	E5	4	23	56	511	24	42	515	15	26	441
1896	E3	2	57	101	1149	40	92	1012	17	49	985
1968	E6	2	8	11	188	7	15	175	1	11	151

**Table 5:** Quantile regression models for the median of the natural logarithm of emission compounds for each testing point. The values represent $\exp(\hat{\beta })$ where $\hat{\beta }$ stands for the estimated regression coefficients. All regression coefficients are significant with p<0.001.

	Testing Point								
	1	1a	1b	2	2a	2b	3	3a	3b
Inter -cept	85.703	73.245	26.518	164.948	141.144	104.001	1798.837	1697.458	1609.175
Eng. disp. ($\times 10^{3}$ cm${}^{3}$)	1.032	1.025	1.048	1.029	1.041	1.023	1.033	1.032	1.037
Mileage ($\times 10^{5}$ km)	1.061	1.047	1.039	1.051	1.042	1.068	1.050	1.051	1.048
E3 STD	0.480	0.483	0.507	0.474	0.485	0.497	0.496	0.495	0.487
E4 STD	0.238	0.243	0.251	0.239	0.236	0.252	0.247	0.247	0.243
E5 STD	0.172	0.174	0.172	0.171	0.171	0.177	0.177	0.177	0.174
E6 STD	0.078	0.076	0.081	0.077	0.076	0.081	0.079	0.079	0.077

**Table 6:** Quantile regression models for the 75% quantile of the natural logarithm of emission compounds for each testing point. The values represent $\exp(\hat{\beta })$ where $\hat{\beta }$ stands for the estimated regression coefficients. All regression coefficients are significant with p<0.001.

	Testing Point								
	1	1a	1b	2	2a	2b	3	3a	3b
Inter -cept	98.221	77.504	43.530	186.047	153.052	120.426	1990.715	1838.081	1745.733
Eng. disp. ($\times 10^{3}$ cm${}^{3}$)	1.030	1.030	1.029	1.028	1.034	1.026	1.028	1.037	1.033
Mileage ($\times 10^{5}$ km)	1.049	1.053	1.014	1.054	1.053	1.046	1.047	1.054	1.044
E3 STD	0.494	0.494	0.501	0.481	0.493	0.506	0.499	0.486	0.490
E4 STD	0.243	0.248	0.243	0.242	0.246	0.250	0.249	0.243	0.249
E5 STD	0.174	0.181	0.178	0.171	0.176	0.179	0.179	0.174	0.179
E6 STD	0.077	0.080	0.077	0.077	0.079	0.080	0.080	0.078	0.079

**Table 7:** Quantile regression models for the 90% quantile of the natural logarithm of emission compounds for each testing point. The values represent $\exp(\hat{\beta })$ where $\hat{\beta }$ stands for the estimated regression coefficients. All regression coefficients are significant with p<0.001.

	Testing Point								
	1	1a	1b	2	2a	2b	3	3a	3b
Inter -cept	112.980	90.018	48.772	212.902	172.872	140.140	2128.675	1957.509	1818.451
Eng. disp. ($\times 10^{3}$ cm${}^{3}$)	1.030	1.033	1.034	1.026	1.027	1.032	1.031	1.032	1.027
Mileage ($\times 10^{5}$ km)	1.037	1.038	1.046	1.038	1.050	1.027	1.042	1.045	1.049
E3 STD	0.496	0.487	0.495	0.490	0.493	0.510	0.497	0.496	0.496
E4 STD	0.241	0.245	0.247	0.249	0.251	0.243	0.248	0.248	0.249
E5 STD	0.174	0.179	0.175	0.171	0.177	0.175	0.180	0.178	0.180
E6 STD	0.076	0.077	0.078	0.078	0.079	0.078	0.080	0.079	0.080

**Table 8:** The proportions (%) of vehicles with a real value of emission compounds above the estimate of the 75% quantile and 90% quantile for each testing point, based on ECS.

	Engine Condition State						
TP	1 (n = 553)	2 (n = 596)	3 (n = 1786)	4 (n = 1795)	5 (n = 1810)	1–2 (n = 1149)	3–5 (n = 5391)
1	6.5/0.0	43.3/19.1	70.0/49.9	89.4/70.4	100.0/89.0	25.6/9.9	86.5/69.9
1a	4.7/0.0	46.5/19.1	74.4/53.2	96.3/76.7	100.0/96.3	26.4/9.9	90.3/75.5
1b	18.4/2.7	33.2/18.1	39.4/28.6	44.2/34.9	53.2/43.1	26.1/10.7	45.6/35.6
2	5.2/0.0	43.0/17.3	66.4/46.3	86.1/68.4	99.6/86.7	24.8/9.0	84.1/67.2
2a	4.5/0.0	47.0/23.5	75.4/53.4	96.3/76.1	100.0/95.8	26.5/12.2	90.6/75.2
2b	7.1/0.0	43.5/19.1	69.8/47.3	91.6/72.1	100.0/91.7	25.9/9.9	87.2/70.5
3	0.0/0.0	49.8/20.0	99.4/79.1	100.0/100.0	100.0/100.0	25.8/10.4	99.8/93.1
3a	0.0/0.0	48.8/21.6	99.9/94.2	100.0/100.0	100.0/100.0	25.3/11.2	100.0/98.1
3b	0.0/0.0	48.8/21.5	100.0/99.0	100.0/100.0	100.0/100.0	25.3/11.1	100.0/99.7

**Table 9:** The distribution of the vehicles (absolute frequencies) based on the number of testing points in which the vehicles from the second test set had value emission compound values higher than Q75 and Q90 (with respect to their ECS).

Number of Testing Points above Q75/Q90	Q75		Q90	
	ECS (1–2)	ECS (3–5)	ECS (1–2)	ECS (3–5)
0	344	0	633	0
1	197	0	169	1
2	99	0	198	17
3	128	1	92	101
4	158	31	44	303
5	130	166	11	645
6	56	476	2	912
7	30	898	0	1267
8	6	2200	0	1510
9	1	1619	0	635

## Data Availability

The data presented in this study are available on request from the corresponding author. The data are not publicly available due to ongoing research in area of interest.
